# Tailored implementation of cardiovascular risk management in general practice: a cluster randomized trial

**DOI:** 10.1186/s13012-016-0460-0

**Published:** 2016-08-11

**Authors:** Jan van Lieshout, Elke Huntink, Jan Koetsenruijter, Michel Wensing

**Affiliations:** 1 IQ healthcare, Radboud Institute for Health Sciences, Radboud university medical center, Nijmegen, The Netherlands; 2 Department of General Practice and Health Services Research, University Hospital Heidelberg, Vossstrasse 2, 69115 Heidelberg, Germany

**Keywords:** Implementation, Tailored interventions, Cardiovascular disease, Risk factors, Primary care, Lifestyle, Counselling, Randomized controlled trial, Cluster randomization

## Abstract

**Background:**

Counselling on health-related lifestyles is key to the prevention and management of chronic diseases. After comprehensive study of determinants of its delivery in general practice and strategies to improve, we composed a tailored improvement program, which included communication skills training, online patient information, and a clinical protocol for managing depressive symptoms. Our aim was to assess the effectiveness of this program on professional performance and outcomes in cardiovascular patients.

**Methods:**

A two-arm cluster randomized trial in 34 general practices involving 34 nurses was conducted. The primary outcome was an aggregated score of a positive score on lifestyle counselling delivered and an appropriate action on depressive symptoms. Secondary outcomes included the various elements of the primary outcome, vascular risk factors (extracted from patient records), and patient-reported lifestyle behaviors. Data were collected from medical records and a written survey among included patients.

**Results:**

A sample of 1782 patients with recorded cardiovascular disease or high cardiovascular risk was available at follow-up at 6 months. No impact on the primary outcome was found; lifestyle counselling was recorded in a minority of patients (11.4 % in the intervention group and 10.3 % in the control group). An effect was found on a secondary outcome: patients’ physical activity level increased (*B* 0.18; 95 % CI 0.02–0.35) on a seven-point scale.

**Conclusions:**

The tailored improvement program showed no effect on the primary outcome. This challenges the methodology of tailoring. More involvement of the targeted health care professionals might offer ways to develop more effective implementation programs. Physical activity might be the lifestyle issue that can be more easily changed than smoking or dietary habits.

**Trial registration:**

Nederlands Trial register NTR4069

## Background

Atherosclerosis-related disease is increasingly prevalent as a result of aging populations, unhealthy lifestyles, and survival of patients with potentially lethal cardiovascular diseases (CVD) after effective treatment. Clinical guidelines on cardiovascular risk management (CVRM) provide clear recommendations on risk assessment and monitoring, health-related lifestyles, and preventive medication [1]. Nevertheless, an international study in general practice, which is the setting where many of these recommendations have to be implemented, showed room for improvement of current practice [2, 3].

Tailored implementation seems a promising way to improve CVRM. It is an approach in which determinants of practice are prospectively identified, followed by systematic matching of strategies to the identified factors [4]. A systematic review of 32 trials confirmed the positive impact of tailored implementation interventions, but also highlighted the uncertainty on the usefulness of different methods for tailoring [5]. As part of a large, international study of tailored implementation, the Tailored Implementation for Chronic Diseases (TICD) project, we adopted these strategies to CVRM in the Netherlands. In an interview study, primary care professionals, patients, and other stakeholders emphasized the challenges of counselling patients on health-related lifestyles, medication adherence, and self-management [6–8]. We developed and evaluated a tailored implementation program to address these challenges, based on a comprehensive empirical analysis of determinants of delivering recommended CVRM and suggestions for interventions. The aim of the present study was to determine the effectiveness of the tailored implementation program on professional performance and outcomes in cardiovascular prevention compared to usual care in general practice. In the Netherlands, patient education and counselling of cardiovascular patients is mainly provided by practice nurses [9], so they were the primary target of the improvement program.

## Methods

### Study design

An open-label, two-arm, cluster randomized trial was conducted in the years 2013 and 2014 in the Netherlands [10]. The study was part of the international TICD project [11]. We performed block randomization of the participating general practices, stratifying for practice size (one general practitioner versus two general practitioners or a group practice) and practice location (rural versus urban), using a computer program that was handled by an independent researcher. The Medical Ethics Committee of Arnhem-Nijmegen waved approval for the study (file 2013/229).

### Samples

**Fig. 1:**
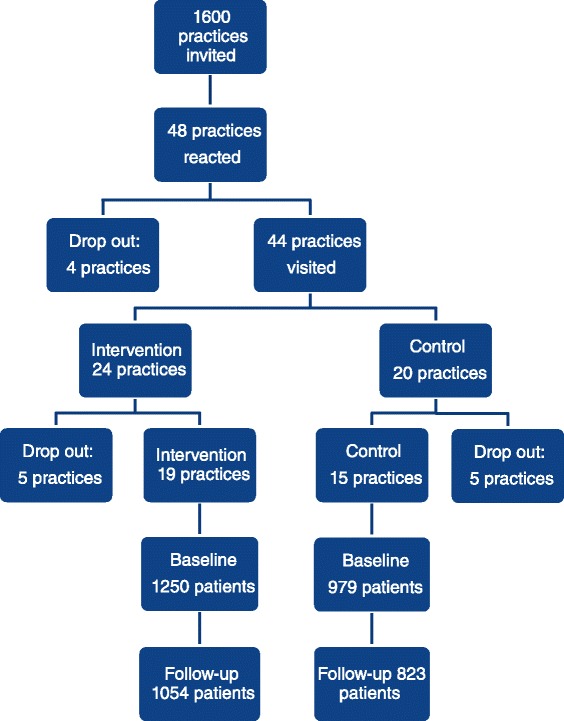
Flow chart of the study

A random sample of general practices in seven geographical areas in the Netherlands was invited to participate in the study, resulting in a sample of 34 practices at baseline. Figure 1 presents the flow of participants through the study. In the participating practices, two samples of patients were approached for participation in the study: patients at high cardiovascular risk and patients with established CVD. These high risk patients have an estimated 10-year risk score of 20 % or higher for morbidity and mortality due to CVD. Patient selection was based on the following International Classification of Primary Care (ICPC) codes: K74-K76, K85-K92, K99.1, and T93. Patients had to be adults aged 18 or older, have a high risk of CVD (but no known CVD) or established CVD, and capable of providing informed consent. Exclusion criteria were diabetes mellitus, pregnancy and lactation, terminal illness, cognitive impairment, and poor language skills.

### Implementation program

A tailored implementation program was developed in a systematic, stepwise process. We have reported on the various steps in this developmental process before [6–8, 12]. First, prevailing clinical guidelines [13, 14] and clinical audit data were analyzed to define the following interrelated targets for improvement: systolic blood pressure (SBP) <140 mmHg in patients with established CVD or in patients at high risk for CVD; low-density lipoprotein (LDL) cholesterol <2.5 mmol/l in patients with established CVD or in patients at high risk for CVD; promote lifestyle changes in patients with (high risk for) CVD; create a risk profile for patients with chronic kidney disease.

Then, an interview study was done, involving physicians, nurses, and patients, which identified 139 plausibly important determinants of practice (“barriers or enablers of implementation”). Of this list, a set of 11 determinants was selected based on importance and changeability as judged by the research team and used for subsequent steps [8, 12]. Subsequently, group interviews with different stakeholders and patients generated 181 suggested strategies for implementation, which were perceived to address the selected set of 11 most relevant determinants [6].

The research team discussed the large number of strategies suggested. Physicians and nurses in Dutch general practices expressed an interest in continued training of their motivational counselling skills, although research seemed to suggest little impact [15, 16]. They also expressed an interest in using online information tools for patients more actively in their patient counselling. As depressive symptoms are an important moderator of patient counselling, the recommendations suggested addressing depressive symptoms first, before focusing on health-related lifestyles or adherence to preventive drug therapy. Considering feasibility and potential impact, the research team selected the following implementation strategies for this trial: structured feedback by professional trainers to practice nurses on their motivational interviewing skills in practice (“refresher training”); access to an online educational program on CVRM, which was developed by the Dutch College of General Practitioners; written guidance on relevant e-health options for patients, emphasizing www.thuisarts.nl and hartenvaatgroep.nl, and a planned Twitter consultation hour for patients; and a flow chart for dealing with depressive symptoms in cardiovascular patients. The last item was an elaboration of the recommendation in the guideline on cardiovascular risk management “to consider relevant co-morbidities.” It suggested to treat major depression before giving any lifestyle advice and to promote physical exercise rather than any other lifestyle in patients with mild depressive symptoms. A detailed description of the implementation program has been published in the study protocol [10]. General practices in the control arm of the study provided usual care and were offered a delayed intervention after the follow-up measurements had been completed.

### Measures

Two waves of measurements were done in each practice: at baseline and at follow-up 6 months later. Data for this paper were collected from computerized patient records in the general practices and from a written survey in a cohort of patients. Descriptive characteristics of participating practices and practice nurses were recorded with a structured questionnaire, which was completed by practice nurses.

A modified version of the validated EPA Cardio abstraction tool was applied to collect data from patient records in participating general practices [17]. We collected data on recorded patient counselling on CVD-related lifestyle and on the presence of a record of depressive symptoms in the intervention period and actions related to that record. We recorded the latest value if any of the SBP, the LDL cholesterol level, the BMI and the smoking status in the intervention period, and the latest value before the intervention period started with a retrospective time window of 1 year. Furthermore, we collected data on the presence of the following co-morbidities: asthma, COPD, and rheumatoid arthritis.

Patients received a paper-based questionnaire at inclusion and at the end of the intervention period of 6 months. We asked for their highest level of education as a proxy for social economic status. Furthermore, we assessed the presence of depressive symptoms using the Patient Health Questionnaire-9 (PHQ-9) [18]. Scores of 5, 10, 15, and 20 represent cut points for mild, moderate, moderately severe, and severe depression, respectively. Based on these cut points, we considered a score up to 5 as no depressive symptoms, 6 to 15 as mild depressive symptoms, and a score above 15 as severe depressive symptoms in our description of the patient sample.

Finally, we used the Rapid Assessment of Physical Activity (RAPA; 9 items) [19] to assess patient’s physical activity level and the shortened Rapid Eating and Activity Assessment (REAP-S; 12 items) [20] to assess dietary habits.

### Primary and secondary outcomes

In our international working group, coordinating the trials in the countries collaborating, we decided to provide a common primary outcome measure across all trials based on the performance of the health care professional targeted by the interventions. The pre-defined primary outcome referred to the professional performance of practice nurses and reflected adoption of recommendations for personalized counselling and education of CVRM patients. We created a dichotomous score for measurement in each patient, reflecting adequate or inadequate performance. We considered practice nurses’ professional performance to be adequate when at least one of the following two conditions was met: (1) there is a record in the patient’s record that the patient has received advice on at least one lifestyle item as specified in the prevailing guidelines of CVRM: diet, smoking, or physical exercise. Also, at least one target for improving an aspect of lifestyle is recorded. When a patient has a perfect lifestyle, then that will be recorded. (2) There is a notation in the patient’s record that the patient has none, mild, or severe depressive symptoms and that the patient has been referred to E-health, a physical exercise group or depression treatment, respectively. If there was no record, we considered that there was no personalized counselling or education to the patient.

As secondary outcomes, we recorded the various elements contributing to the composite primary outcome: the health care received by each patient (counselling on lifestyle with personal goal setting, referral to a physical exercise group, referral for depression treatment). Furthermore, as secondary outcomes, we documented blood pressure (SBD <140), cholesterol levels (LDL <2, 5), body mass index (BMI <25), smoking status (yes/no), food intake (REAPS, range 1–3), and physical exercise (RAPA, range 1–7).

### Statistical power

The study was powered to detect a 15 % difference on the primary outcome. For sample size calculation, we used a web-based program [21], and based on previous research, we assumed an intra-cluster correlation coefficient (ICC) of 0.05 [22, 23], alpha of 0.05, and a power of 0.80. The calculation indicated that 450 patients per group (high risk or established CVD) would be needed (15 patients at high risk for CVD and 15 patients with established CVD per cluster, sampled in 30 practices). Assuming high risk patients often only visit the practice once a year and that half of the CVD patients receive specialist care, we doubled the numbers of patients for inclusion. Furthermore, to allow for loss to follow-up, we enlarged the inclusion with another 30 %.

### Data analysis

Data were analyzed using SPSS (version 20, IBM Corp.) and MLWIN (version 2.28). For all primary statistical tests, two-sided hypothesis testing with an alpha level of 0.05 was applied. All data analyses were based on “intention to treat.” For assessing effects on the outcomes, the intervention and control group were compared regarding each of the primary and secondary outcomes. In the study protocol, we planned to perform a chi-square test to assess the primary outcome. However, we reconsidered this in favor of a multilevel regression analysis to meet the highest analytical standards. The primary outcome was based on measurements at follow-up only and therefore a two-level model was used (patients nested in practices). We entered group allocation (intervention/control) at practice level and controlled for the following patient characteristics: age, sex, education (low, medium, high), depressive symptoms (none, mild, severe), and co-morbidities (presence of asthma, COPD, and/or rheumatoid arthritis). To test for differences between the high risk and CVD patients, we entered an interaction term with group allocation (control/intervention) and patient group (high risk/CVD). For the secondary outcomes with baseline and follow-up measures, we used a three-level multilevel regression model with measurements nested in patients, and patients were nested in practices. Therefore, we constructed the data in a long format and by including an extra level, we controlled for differences in individual patients at baseline. We had planned to assess the cardiovascular risk score in the high-risk patient group and changes in these scores, but we had to refrain from these analyses as not feasible.

## Results

We invited 1600 practices to participate in our program; initially, 48 responded that they wanted to participate. Before group allocation and an introductory practice visit, 4 practices withdrew, and after the initial practice visit, another 10 practices withdrew, 5 from both the intervention and the control group. So, 34 practices entered the study and included patients (Fig. 1). Two practices in the intervention arm had two practices nurses, all participating and being instructed; in both the intervention arm and the control arm, one practice nurse worked in two practices. No practices were lost to follow-up. One practice nurse dropped out at the end of the intervention period due to the fact that she started to work in another practice but even from that practice, we were able to retrieve data at the end of the intervention period.

In total 2229 patients (41.8 % of those invited) gave informed consent for the study in the baseline patient questionnaire. In all groups (intervention and control, high risk, and established CVD), men were about twice as numerous as women. The high risk group patients were about 75 years old, and the CVD patients just under 70. In total, 75 % of the patients had no depression, about 23 % had mild depressive symptoms and 2 % had a severe depression, based on the PHQ-9 questionnaire at baseline. Tables 1 and 2, respectively, display some practice characteristics and patient characteristics at baseline.

**Table 1:** Practice and practice nurse characteristics

	Intervention group (*n* = 19)	Control group (*n* = 15)
	19 practices with 20 practice nurses	15 practices with 14 practice nurses
Practice characteristics		
Single-handed practice	10	9
Duo/group practice	9	6
Rural area	10	6
Urban area	9	9
Practice nurse characteristics		
Mean age in years	42	43
Mean number of years experience as practice nurse	12	11
Mean number of hours previous training of motivational interviewing skills	14.7	14.8

**Table 2:** Description of patient sample at baseline

	Intervention group (*n* = 1250)			Control group (*n* = 979)		
	Total	Patients with high cardiovascular risk	Patients with cardiovascular disease	Total	Patients with high cardiovascular risk	Patients with cardiovascular disease
Women (%)	35.1	32.1	38.4	34.9	31.7	38.5
Mean age (SD)	72.6 (9.7)	75.1 (6.4)	69.6 (11.8)	71.6 (9.7)	74.0 (6.5)	68.9 (11.7)
Education low (%)	39.8	38.3	41.6	43.0	42.0	44.0
Education medium (%)	31.7	30.7	32.8	32.1	30.0	34.5
Education high (%)	28.5	31.0	25.6	24.9	28.0	21.5
Hypertension (%)	59.9	67.7	51.1	59.2	65.6	52.0
Hypercholesterolemia (%)	18.4	16.6	20.4	20.2	16.1	24.8
BMI >25 (%)	30.2	29.4	31.3	30.6	29.3	32.4
Recorded smokers (%)	10.4	7.2	13.8	10.5	8.9	12.2
Other chronic disease (%)	14.8	12.9	16.9	14.5	13.0	16.0
No depression (%)	74.4	79.2	68.8	75.6	85.4	64.9
Mild depression (%)	23.6	19.1	28.7	22.7	13.6	32.7
Severe depression (%)	2.0	1.7	2.5	1.7	1.0	2.4

### Primary outcome

We found no effect of our intervention on the primary outcome of this study (see Table 3). A record proving adequate practice nurse performance was present in 11.4 % of the patients in the intervention practices and in 10.3 % of the patients in the control group. There was in only six patients a record of depressive symptoms. The element of this composed primary outcome measure related to physical exercise advice was more often recorded in the intervention group (6.8 versus 3.7 %), though in multilevel regression analysis, this proved to be non-significant. The other components, too, showed no differences.

**Table 3:** Primary outcomes: medical audit data on patient counselling

	Intervention arm	Control arm	OR (95 % CI)
	(*n* = 995 patients)	(*n* = 787 patients)	
	% (*n*)	% (*n*)	
Number of patients who received recommended counselling (=primary outcome measure)	11.4 (113)	10.3 (81)	1.11 (0.56–2.21)
In subgroup of patients with high cardiovascular risk	11.9 (63)	12.0 (47)	1.00 (0.42–2.38)
In subgroup of patients with cardiovascular disease	10.7 (50)	8.6 (34)	1.27 (0.68–2.38)
Recorded physical exercise advice/goal	6.8 (68)	3.7 (29)	1.85 (0.68–5.04)
Recorded stop-smoking advice	1.4 (14)	1.5 (12)	1.07 (0.43–2.64)
Recorded diet advice	7.9 (78)	7.8 (61)	0.95 (0.39–2.31)
Recorded goal for lifestyle change in patients with no record of depressive symptoms (99.6 %)	11.3 (112)	10.2 (80)	1.10 (0.55–2.17)
Recorded physical exercise advice or referral in mild depressed patients (relates to 6 patients)	0.1 (1)	0.0 (0)	NA
Recorded depression treatment or referral in severe depressed patients (relates to 1 patient)	–	0.1 (1)	NA

*NA* not assessed

### Secondary outcomes

Regarding secondary outcomes, we found that physical exercise showed a significant improvement in the intervention group compared to the control group (see Table 4). The RAPA score improved on a scale from 1 to 7 from 4.8 to 4.9; in the control group, the activity diminished reflected in a score diminishing from 4.9 to 4.8 (effect size *B* = 0.18 (0.02–0.35), *p* < 0.05). On the other cardiovascular risk factors assessed (SBP, LDL cholesterol, smoking status, BMI, and diet), the improvement program had no significant effect. We found no difference in the effect of the intervention between the high cardiovascular risk group and the group with established CVD. However, CVD patients had their LDL cholesterol and SBP level more often on target (OR 3.8, 95 % CI 2.9–5.1 and OR 1.5, 95 % CI 1.2–1.8, respectively).

**Table 4:** Secondary outcomes: risk factors (medical audit data and patient questionnaires)

	Intervention arm		Control arm		
	Baseline	Follow-up	Baseline	Follow-up	*B*/OR (95 % CI)
Physical exercise (RAPA, 1–7)	4.8 (1.60)	4.9 (1.52)	4.9 (1.59)	4.8 (1.53)	*B* 0.18* (0.02–0.35)
Diet (REAP-S, 1–3)	2.2 (0.38)	2.3 (0.37)	2.2 (0.38)	2.2 (0.37)	*B* 0.03 (0.00–0.07)
Smoking (%)	10.3	10.4	12.5	10.5	OR 1.11 (0.68–1.82)
BMI <25 (%)	29.5	30.2	26.3	30.6	OR 0.84 (0.48–1.46)
LDL <2.5 (%)	30.5	32.3	26.6	31.2	OR 0.85 (0.53–1.38)
SBP <140 (%)	57.6	56.9	57.9	57.1	OR 1.03 (0.72–1.48)

*B* regression coefficient, *OR* odds ratio

*p < 0.05

## Discussion

The tailored implementation program had no overall effect on counselling of cardiovascular patients (the primary outcome of the trial). However, we found an effect on one secondary outcome: patients’ physical activity level increased. The latter finding may suggest that the messages on physical exercise, as recommended for patients with mild depressive symptoms, were picked up by practice nurses and applied in daily practice. Nevertheless, overall, we conclude that the tailored implementation program was not effective.

There are several alternative explanations for the lack of effects. In process evaluations, we will report on the outcomes of interviews and questionnaires with practice nurses and a sample of patients, and on scoring of knowledge and motivational interviewing skills [10, 24]. In general, we can hypothesize that failure of effectiveness might be due to lack of relevance of determinants or strategies, wrong prioritizing, or inadequate intervention delivery. First, identified determinants of practice may be less relevant than perceived by stakeholders or proposed interventions may be less helpful than expected. For instance, studies in general practice seemed to suggest little effect of motivational interviewing (MI) skills training [15, 16]. In the developmental phase of our study, we held focus group discussions with various stakeholders [7, 8] and interviewed them about suggestions for interventions to address previous identified determinants of practice. All stakeholder groups suggested motivational interviewing training and participating primary care providers appreciated such training. Koelewijn et al. in the IMPALA study offered an improvement program providing MI training to practice nurses [15]. Patients included were at high cardiovascular risk without established CVD. They could not prove the program to be effective. Jansink developed and tested a program including MI courses for diabetic nurses [16]. This program, too, showed no improvement in their main outcomes. These findings challenge the use of interviews and surveys with stakeholders in tailored implementation as well as the usefulness of motivational interviewing in the targeted patient populations.

Second, numbers of both determinants of practice and suggestions for improvement were high, so it is possible that in our tailoring procedures, we did not prioritize the right determinants in terms of importance and changeability or the right interventions considering feasibility and impact. For instance, we might have better focused on practice nurses’ views on effective interventions, as they were the primary receivers of the tailored implementation program. Involvement of practice nurses in selecting strategies in the development of the multifaceted program might have been another method to assure adequate choices in this phase.

Third, we may have chosen interventions adequately, but not delivered them in the required intensity. For instance, a short training session with feedback on two patient contacts may have been insufficient to effectively improve counselling skills. Some of the information technology tools were innovative, so it may be too early to implement these effectively or more time might be needed. Finally, the presence of a practice nurse with some education in counselling techniques was an inclusion criterion for practices as we intended to provide a refresher course instead of a complete course. So, the practice nurses in the control group were somehow skilled thus reducing the potential for improvement by the tailored program.

Another explanation of the poor results may relate to the level of tailoring. In our international project, we had clearly defined and separate steps in the developmental phase of the intervention program. For that reason, we collected information on determinants of practice and suggestions in groups of stakeholders. These groups comprised health care professionals finally targeted but not those from the practices that participated in our trial. In another setting, the tailoring could be on a higher level, i.e., on the level of the practices or health care professionals participating with adjustments. Then, we would have researched the determinants within the practices participating and searched for strategies specifically suitable and tailored per practice. In the process evaluation, we will report on the practice nurses’ opinion on the determinants addressed and the interventions offered [21].

We found an overall low score on practice nurse performance in our primary outcome as measured by the results of the chart audit. The maximum feasible score for this outcome is unknown but definitely not 100 %. In general, the high risk patients pay a CVRM-related visit to the practice only once a year and a proportion of the patients with established CVD is treated in secondary care. Nevertheless, considering the fact that a large group of about half of the patients did not visit the practice for CVRM in the intervention period and was not exposed to the intervention, the low scores still suggest room for improvement. Poor documentation may add to the low score on the primary outcome. In the introduction and instruction of the project, we emphasized the importance of good registration. The relevance of making record notes in the explanation of the trial results will be part of the process evaluation.

The positive effect on one of our secondary outcome measures relates to physical activity. We advised to refer patients with mild depressive symptoms to physical activities as these are beneficial for both the cardiovascular risk profile and depressive symptoms. As such, in our program delivery, we had extra attention for physical exercise, more than for other lifestyle issues. Without a record of depressive symptoms, more patients had a record of advice on physical activity with a personal goal. Apparently, this lifestyle issue gained more attention in the intervention group. Recording advice, a process indicator, improved non-significant. The related patient outcome “physical activity” improved significantly which makes it more plausible that this effect is not related to chance.

The study was designed to enhance internal validity as well as reflect routine clinical practice, but it also had a number of limitations. Interviews with stakeholders were used to identify determinants of practice and suggestions for interventions, but it is difficult to assess the validity of this method. Stakeholder involvement in the design of interventions may in fact have served a different purpose, which is enhanced acceptability of the program for the targeted health care providers. The generalizability of findings may be limited by the low recruitment rate, although it seems unlikely that more effects would have been achieved in non-participants. Surveys were planned at baseline and 6 months later, but this was only partly achieved. Due to practical constraints, patients’ questionnaires were sent out up to 2 months later. Note however that most outcomes, including the primary outcome, were based on chart audits taking into account the start date of the intervention period. As the study was a pragmatic trial, we cannot rule out the possibility that external influences have had impact on the outcomes. In the field of cardiovascular primary care, changes in the reimbursement and quality management may have impacted on practices in both intervention and control arms of the study diluting possible effects of our program.

## Conclusions

For tailoring an intervention, we recommend including a systematic method for assessment and prioritization of determinants of practice and suggested implementation interventions. The program may benefit from more attention for the targeted group, the practice nurses in our program. On the basis of the findings of this trial, we cannot recommend broad implementation of the tested improvement program. In a process evaluation of the trial, we will explore the impact of interventions on the addressed determinants of practice [10]. This will provide further insight into the validity of the interview methods, which were used in the development of the implementation program. Our process evaluation and future research may elucidate whether attention on physical activity could be a key target for future programs to improve cardiovascular prevention. Future studies should explore alternative methods for tailored implementation, such as theory-orientated approaches or different stakeholder involvement methods.

## Abbreviations

BMI, body mass index; CVD, cardiovascular disease; CVRM, cardiovascular risk management; ICPC, International Classification of Primary Care; LDL, low-density lipoprotein; SBP, systolic blood pressure; TICD, Tailored Implementation for Chronic Diseases
